# Intranasal Insulin Prevents Anesthesia-Induced Cognitive Impairment and Chronic Neurobehavioral Changes

**DOI:** 10.3389/fnagi.2017.00136

**Published:** 2017-05-10

**Authors:** Yanxing Chen, Chun-Ling Dai, Zhe Wu, Khalid Iqbal, Fei Liu, Baorong Zhang, Cheng-Xin Gong

**Affiliations:** ^1^Department of Neurology, The Second Affiliated Hospital, School of Medicine, Zhejiang UniversityHangzhou, China; ^2^Department of Neurochemistry, Inge Grundke-Iqbal Research Floor, New York State Institute for Basic Research in Developmental DisabilitiesNew York, NY, USA; ^3^Department of Cell Biology and Genetics, School of Basic Medicine, Hubei University of Science and TechnologyXianning, China

**Keywords:** Alzheimer’s disease, cognitive impairment, general anesthesia, insulin, intranasal administration, sevoflurane, postoperative cognitive dysfunction, propofol

## Abstract

General anesthesia increases the risk for cognitive impairment post operation, especially in the elderly and vulnerable individuals. Recent animal studies on the impact of anesthesia on postoperative cognitive impairment have provided some valuable insights, but much remains to be understood. Here, by using mice of various ages and conditions, we found that anesthesia with propofol and sevoflurane caused significant deficits in spatial learning and memory, as tested using Morris Water Maze (MWM) 2–6 days after anesthesia exposure, in aged (17–18 months old) wild-type (WT) mice and in adult (7–8 months old) 3xTg-AD mice (a triple transgenic mouse model of Alzheimer’s disease (AD)), but not in adult WT mice. Anesthesia resulted in long-term neurobehavioral changes in the fear conditioning task carried out 65 days after exposure to anesthesia in 3xTg-AD mice. Importantly, daily intranasal administration of insulin (1.75 U/mouse/day) for only 3 days prior to anesthesia completely prevented the anesthesia-induced deficits in spatial learning and memory and the long-term neurobehavioral changes tested 65 days after exposure to anesthesia in 3xTg-AD mice. These results indicate that aging and AD-like brain pathology increase the vulnerability to cognitive impairment after anesthesia and that intranasal treatment with insulin can prevent anesthesia-induced cognitive impairment.

## Introduction

The world population is growing old with the increase in life expectancy. As a result, the number of aged individuals undergoing surgical procedures is increasing. The elderly patients are known to be at an increased risk of postoperative cognitive dysfunction (POCD; Hudson and Hemmings, 2011). POCD is a well characterized syndrome of prolonged impairment of cognition, including memory, intellectual ability and executive function, which occurs weeks to months after surgery (O’Brien et al., 2017). General anesthesia is a major contributor of POCD. Anesthesia may also increase the risk for cognitive impairment and Alzheimer’s disease (AD), especially in the elderly (Seitz et al., 2011; Chen P.-L. et al., 2014; Patel et al., 2016). Population-based studies suggest that the elderly and other vulnerable individuals who are already cognitively impaired before surgery may be at increased risk of dementia (Chen C.-W. et al., 2014; Chen P.-L. et al., 2014; Patel et al., 2016; Sprung et al., 2016).

Animal studies have shed some light on the possible molecular mechanisms involved in anesthesia-induced cognitive impairment. Anesthesia is found to exacerbate the formation of extracellular amyloid plaques and intraneuronal neurofibrillary tangles, the two major neuropathological hallmarks of AD in mice (Perucho et al., 2010; Run et al., 2010). It also increases the generation, oligomerization and accumulation of Aβ, the major component of amyloid plaques (Yang and Fuh, 2015). Anesthesia-induced hypothermia exacerbates tau hyperphosphorylation and subsequently neurofibrillary degeneration (Planel et al., 2007; Run et al., 2010). Under normothermic condition, anesthesia can also induce hyperphosphorylation of tau in animal models (Run et al., 2009; Whittington et al., 2011; Le Freche et al., 2012), indicating that anesthesia may promote tau hyperphosphorylation and tau pathology through multiple pathways. Furthermore, anesthetics are found to disturb blood-brain-barrier permeability, promote neuroinflammation and induce neuronal apoptosis (Mena et al., 2010; Yang and Fuh, 2015). Thus, general anesthesia may induce pathological changes in the brain which may contribute to the cognitive impairment observed post operation in clinic. We recently found that general anesthesia disturbs brain insulin signaling and induces abnormal hyperphosphorylation of tau in 3xTg-AD mice, a triple transgenic mouse model of AD (Chen et al., 2014b). We also found that daily intranasal administration of insulin for 7 days can prevent these anesthesia-induced brain changes (Chen et al., 2014b), as well as the anesthesia-induced deficit in spatial learning and memory, as determined by Morris Water Maze (MWM) task during 1–5 days after exposure of aged mice to anesthesia (Zhang et al., 2016). However, many important issues concerning the understanding of the role of anesthesia in POCD and its prevention remain to be elucidated. For example, how do aging and AD-like brain pathology impact the vulnerability of mice to anesthesia-induced cognitive impairment? Does anesthesia induce any long-term cognitive impairment besides the short-term memory deficit? Can intranasal insulin also be effective in those with prior pathological conditions? The present study was designed to address these important questions.

In the present study, we exposed mice of different ages and of prior pathological conditions to anesthetics (propofol and sevoflurane) and then investigated their short- and long-term effects on neurobehavioral performance. We also studied the effects of intranasal insulin administered prior to anesthesia on the neurobehavioral changes. We found that intranasal insulin prevented both the short-term cognitive impairments and the long-term neurobehavioral abnormalities observed in 3xTg-AD mice.

## Materials and Methods

### Antibodies and Reagents

Primary antibodies used in this study are listed in Supplementary Table S1. Peroxidase-conjugated anti-mouse and anti-rabbit IgG were obtained from Jackson ImmunoResearch Laboratories (West Grove, PA, USA). The enhanced chemiluminescence (ECL) kit was from Pierce (Rockford, IL, USA). Propofol was purchased from MP Biomedicals (Solon, OH, USA). Insulin (HumulinRU-100) was from Eli Lily (Indianapolis, IN, USA). Other chemicals were from Sigma-Aldrich (St. Louis, MO, USA).

### Animals and Animal Treatments

The breeding pairs of homozygous 3xTg-AD mouse harboring PS1_M146V_, APP_Swe_ and tau_P301L_ transgenes and the wild type (WT) control mouse (a hybrid of 129/Sv and C57BL/6 mice) were initially obtained from Dr. F.M. LaFerla through Jackson Laboratory (New Harbor, ME, USA), and the mice were bred in the animal colony of New York State Institute for Basic Research in Developmental Disabilities. Mice were housed (4–5 female animals per cage) with a 12/12 h light/dark cycle and with *ad libitum* access to food and water. The housing, breeding and animal experiments were in accordance with the approved protocol from the Institutional Animal Care and Use Committee of New York State Institute for Basic Research in Developmental Disabilities, according to the PHS Policy on Human Care and Use of Laboratory animals (revised March 15, 2010).

The 3xTg-AD mice (female, 7–8 months old) and WT mice (female, 7–8 months old and 17–18 months old) used for the present study were habituated to handling for 14 days prior to the experiment. Female mice were used because the female 3xTg-AD mice develop behavioral deficits faster than the male mice (Clinton et al., 2007). Intranasal delivery was carried out manually without anesthesia while the mouse head was restrained in a supine position with the neck in extension, as described (Marks et al., 2009). A total of 1.75 U insulin in 17.5 μl or, as a vehicle control, 17.5 μl 0.9% saline was delivered over both nares alternatively using a 10-μl Eppendorf pipette. The mouse was held for an additional 5–10 s to ensure the fluid was inhaled. The successful nasal delivery by using this approach was confirmed by examining the incorporation of ink in the autopsied brains after intranasal delivery with ink using the same approach (data not shown).

Anesthesia was induced with intraperitoneal (i.p.) injection of propofol (150 mg/kg body weight) dissolved in intralipid or, as a control, the equivalent amount of intralipid and was maintained by inhalation of 2.5% sevoflurane for 1 or 3 h. After awaken from anesthesia, the mice were returned to their home cages for behavioral tests at later dates. A different cohort of female, 7–8-month-old WT mice (*n* = 6) were sacrificed at the end of 1-h anesthesia, and the forebrains were immediately removed, flash frozen in dry ice, and stored at −80°C for Western blots at a later date.

### Western Blot Analysis

Mouse brain tissue was homogenized in pre-chilled buffer containing 50 mM Tris-HCl (pH 7.4), 50 mM GlcNAc, 20 μM UDP, 2.0 mM EGTA, 2.0 mM Na_3_VO_4_, 50 mM NaF, 20 mM Glycero-phosphate, 0.5 mM AEBSF, 10 μg/ml aprotinin, 10 μg/ml leupeptin and 4 μg/ml pepstatin A. Protein concentrations of the homogenates were determined by using the Pierce 660-nm Protein Assay. The samples were resolved by SDS-PAGE and electro-transferred onto Immobilon-P membrane (Millipore, Bedford, MA, USA). The blots were then probed with primary antibodies listed in Supplementary Table S1 and developed with the corresponding horseradish peroxidase-conjugated secondary antibodies and ECL kit (Pierce, Rockford, IL, USA).

### Morris Water Maze (MWM)

Spatial reference learning and memory were evaluated in a water maze adapted from that previously described by Morris et al. (1982). The test was performed in a white pool of 180 cm in diameter filled with water tinted with non-toxic white paint and maintained at room temperature (21 ± 2°C). During training, a platform (14 cm in diameter) was submerged 1 cm below water surface. All mice received four trials per day for four consecutive days. The starting position was randomized among four quadrants of the pool. For each trial, a mouse was allowed 90 s to locate the hidden platform. If a mouse failed to find the platform within 90 s, it was gently guided to it. At the end of each trial, the mouse was left on the platform for 20 s, then dried and returned to its home cage until the next trial. Probe trial was carried out 24 h after the last day of training. During the probe trial, mice were allowed to swim in the pool without the escape platform for 60 s. The latency to reach the platform site (seconds), number of platform location crossings, time in the target quadrant (seconds), distance covered in the target quadrant (centimeters) and swim speed (centimeters/second) were recorded using an automated tracking system (Smart video tracking system, Panlab, Harvard Apparatus). Reversal water maze task was performed the next day following standard water maze. This involved moving the location of the escape platform diagonally, followed by a 3-day acquisition phase, with a probe trial on day 4. The same parameters as for the standard MWM were recorded using the video tracking system.

### Open Field Test

Exploratory activities and anxiety were evaluated by allowing mice to freely explore an open field arena for 15 min. The testing apparatus was a classic open field (i.e., a polyvinyl chloride square arena, 50 × 50 cm, with walls 40 cm high), surmounted by a video camera connected to a computer. Each mouse was placed individually in the arena and the performance was monitored and the time spent in the center and peripheral area and the distance traveled in the arena were automatically recorded by a video tracking system (ANY-Maze version 4.5 software, Stoelting Co., Wood Dale, IL, USA).

### Novel Object Recognition Test

Mice were tested for one-trial object recognition based on the innate tendency of rodents to differentially explore novel objects over familiar ones in an open field arena, using a procedure modified from a previous description (Sargolini et al., 2003). The procedure consisted of three different phases: a habituation phase, a sample phase and a test phase. Following initial exposure, four additional 10-min daily habituation sessions were introduced to mice for becoming familiar with the apparatus and the surrounding environment. On the fifth day, every mouse was first submitted to the sample phase of which two identical objects were placed in a symmetric position from the center of the arena and was allowed to freely explore the objects for 5 min. After a 15-min delay during which the mouse was returned to its home cage, the animal was reintroduced in the arena to perform the test phase. The mouse was then exposed to two objects for another 5 min: a familiar object (previously presented during the sample phase) and a novel object, placed at the same location as during the sample phase. Data collection was performed using a video tracking system (ANY-Maze version 4.5 software, Stoelting Co.). Object discrimination was evaluated by discrimination index: (time spent exploring the new object/time spent exploring both old and new objects) during the test phase.

### Cued and Contextual Fear Conditioning

The contextual and cued fear conditioning test assesses the ability of mice to learn and remember an association between environmental cues and aversive experiences, which involve amygdala, hippocampus, frontal cortex and cingulate cortex (Fanselow and Poulos, 2005; Xie et al., 2016). Briefly, mice were habituated in the testing room for 1 day before experiment. In a fear-conditioning training session, the mice were habituated to the context for 120 s, and then four tone-shock pairs consisting of a 30-s tone (2000 Hz, 75 dB) co-terminating with a 2-s foot shock at 0.6 mA delivered with a 120-s interval. Afterward, mice remained in the context for 120 s before being returned to their home cage. In the context session on day 2, mice were placed into the same testing chamber without tone or electric foot shock for 5 min to measure freezing response to the context. On day 3, the mice were introduced to the same chamber with altered context. After a 180-s baseline habitation, 180-s tone was delivered without the shock pairing, and then the mice remained in the chamber for 90 s before returned to their home cage. All the data were collected and analyzed with the Freeze Frame and Freeze View system (Coulbourn Instruments, Whitehall, PA, USA).

### Elevated Plus Maze

Elevated plus maze was used to evaluate anxiety/emotionality of the mice. It consisted of four arms (30 × 5 cm) connected by a common 5 × 5 cm center area. Two opposite facing arms were open (OA), whereas the other two facing arms were enclosed by 20-cm high walls (CA). The entire plus-maze was elevated on a pedestal to a height of 82 cm above floor level in a room separated from the investigator. The mouse was placed onto the central area facing an open arm and allowed to explore the maze for a single 8-min session. Between each session, any feces were cleared from the maze, and the maze floor was cleaned with 70% alcohol to remove any urine or scent cues. The number of CA entries, OA entries, and the amount of time spent in CA and OA were recorded by a video tracking system (ANY-Maze version 4.5 software, Stoelting Co.).

### Statistical Analysis

Western blot data were analyzed by unpaired two-tailed *t* tests using Graphpad. For distance traveled in the open field, distance traveled during water maze training, and the freezing percentage during context test and cued tone test of the fear conditioning, repeated measures two-way analysis of variance (ANOVA) with Bonferroni *post hoc* tests were performed using Graphpad. For the rest behavioral measurements, one-way ANOVA with Tukey’s *post hoc* tests or unpaired two-tailed *t* tests were used. In all figures, * indicates *p* < 0.05, and ** indicates *p* < 0.01.

## Results

### Aging and AD-Like Brain Pathology Increase the Vulnerability to Anesthesia-Induced Cognitive Impairment

**Figure 1:**
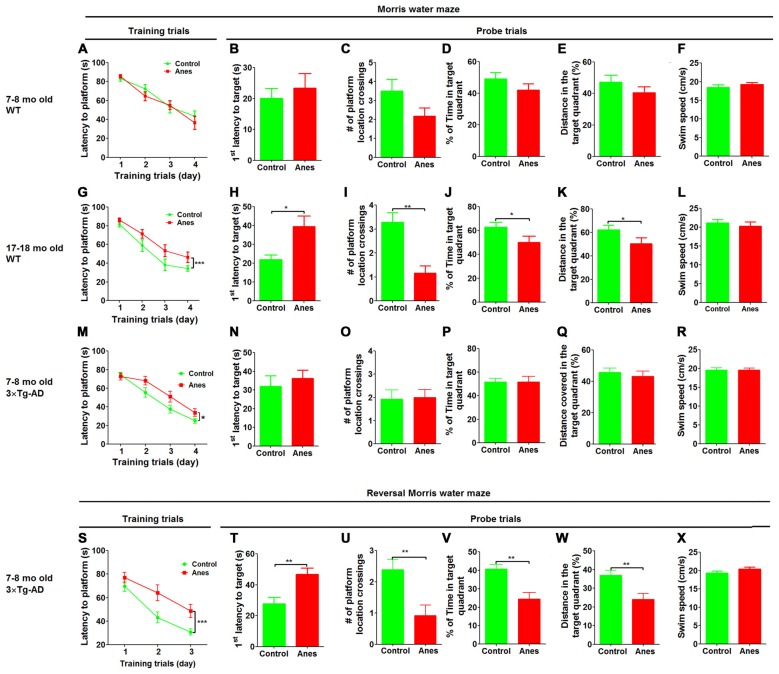
**Effect of anesthesia on spatial learning and memory.** Wild-type (WT) mice of the indicated ages and 7–8-month-old 3xTg-AD mice were trained in Morris Water Maze (MWM) starting 24 h after general anesthesia with propofol and sevoflurane. Control group did not receive anesthesia. **(A,G,M)** Training curves show the latencies to locate the platform during training sessions for four consecutive days (4 trials/day). **(B–F, H–L, N–R)** Probe trial was performed 24 h after the last training trial. The first latency to reach the platform location **(B,H,N)**, the number of the platform location crossings** (C,I,O)**, the percent time the mice stayed in the target quadrant **(D,J,P)**, the distance the mice swam in the target quadrant **(E,K,Q)** and the swim speed **(F,L,R)** of mice during the 60 s probe tests are shown. Reversal MWM test was performed for the 3xTg-AD mice starting on the next day following the probe trial. The training curves **(S)**, as well as the first latency to reach the platform location **(T)**, the number of the platform location crossings** (U)**, the percent time the mice swam in the target quadrant **(V)**, the distance the mice swam in the target quadrant **(W)** and the swim speed **(X)** of the mice during the 60 s probe tests are shown. Data are presented as mean ± SEM (*n* = 10–15 mice per group).

To study whether aging affects the vulnerability to anesthesia-induced cognitive impairment, we investigated spatial reference learning and memory by the MWM task 1 day post anesthesia in adult (7–8 months old) and aged (17–18 months old) mice. The anesthesia was induced by i.p. injection of propofol (150 mg/kg body weight) and maintained by inhalation of sevoflurane (2.5%) for 1 h. We found that the adult mice showed no impairment in spatial reference learning post anesthesia, as indicated by the indistinguishable learning curves during training trials between the anesthesia-treated and control-treated mice (Figure 1A). Probe trial performed 24 h after the last training session showed no significant differences in the latency to the former platform location (Figure 1B), the number of platform location crossings (Figure 1C), the percentage of time spent in the target quadrant (Figure 1D), the percentage of distance the mice swam in the target quadrant (Figure 1E) or the swim speed (Figure 1F). These results suggest that anesthesia induced no detectable reference memory impairment in adult mice. However, when the identical treatment and test were performed in the aged mice, we found a significant impairment in spatial learning post anesthesia, as indicated by the longer times to reach the submerged platform for the anesthesia-treated aged mice than the control-treated aged mice during training sessions (Figure 1G). During the probe trial, the anesthesia-treated aged mice also took longer time to reach the former platform location (Figure 1H), crossed the platform location less number of times (Figure 1I), spent less time in the target quadrant (Figure 1J) and swam a shorter distance in the target quadrant (Figure 1K) than the control-treated aged mice. These two groups of mice had the same swim speed (Figure 1L), which otherwise may interfere the water maze test. These results clearly indicate that anesthesia induced cognitive impairment in the aged mice.

To investigate whether prior AD-like pathology can increase the vulnerability of mice to anesthesia-induced cognitive impairment, we included 7–8-month-old 3xTg-AD mice in the study. These mice are widely used as a transgenic mouse model of AD and develop detectable intraneuronal Aβ accumulation and hyperphosphorylated tau in the brain as well as cognitive impairment at the age of 7–8 months old (Giménez-Llort et al., 2007; Mastrangelo and Bowers, 2008). We found that the anesthesia-treated mice took longer time than the control-treated mice to reach the submerged platform during training sessions (Figure 1M), suggesting an impairment of spatial learning by anesthesia in 3xTg-AD mice. However, probe trial tested 24 h after the last training session showed no significant differences in the latency to reach the platform location (Figure 1N), the number of platform location crossings (Figure 1O), the percentage of time spent in the target quadrant (Figure 1P), the percentage of distance the mice swam in the target quadrant (Figure 1Q) or the swim speed (Figure 1R). We also carried out reversal MWM test to evaluate the flexibility of the spatial learning and memory of these mice. We found a marked impairment in the anesthesia-treated 3xTg-AD mice, as compared to the control-treated mice, during the training phase of the reversal MWM (Figure 1S). Marked impairment of reference memory was also observed in the anesthesia-treated 3xTg-AD mice during the probe trial of the reversal MWM test (Figures 1T–W). Motor function of these mice was not affected by anesthesia (Figure 1X). Because no cognitive impairment was found in the wild type mice of the same age by using the same tests post anesthesia (Figures 1A–F), these results suggest that prior AD-like pathology can increase the vulnerability of mice to anesthesia-induced cognitive impairment.

It is known that anesthesia can induce hyperphosphorylation of tau in the brain, which might contribute to the cognitive impairment (Run et al., 2010). Because no detectable cognitive impairment was observed in the younger adult WT mice post anesthesia, we wondered whether tau hyperphosphorylation was induced in these mice. Western blot analysis of the forebrain homogenates showed tau hyperphosphorylation at all the phosphorylation sites studied except at S214 and S404 in the younger adult mice post anesthesia (Supplementary Figure S1). These results suggest that anesthesia-induced tau hyperphosphorylation itself is insufficient to induce deficits in spatial learning and memory in younger adult WT mice.

### Intranasal Administration of Insulin Prevents Anesthesia-Induced Cognitive Impairment in 3xTg-AD Mice

**Figure 2:**
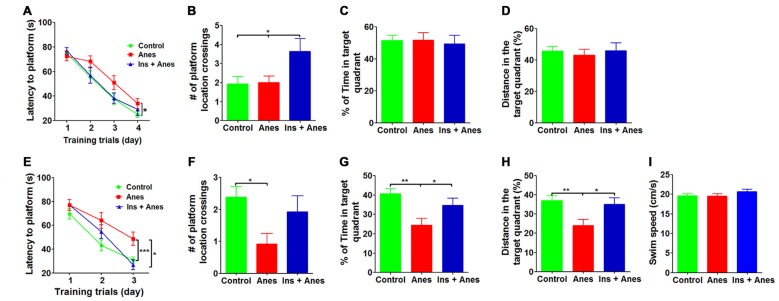
**Effect of intranasal insulin on anesthesia-induced impairment of spatial learning and memory.** The 3xTg-AD mice (7–8 months old, female) received daily intranasal insulin or saline for 3 days, followed by anesthesia with propofol/sevoflurane for 3 h. The mice were then trained in the MWM starting on the following day. **(A)** Training curves show the latency to locate the platform during training sessions for four consecutive days (4 trials/day). **(B–D)** Probe trial was performed 24 h after the last training session. The number of the platform location crossing** (B)**, the percent time the mice swam in the target quadrant **(C)** and the distance the mice swam in the target quadrant **(D)** are shown. Reversal MWM test was performed on the next day following the probe trial. The training curves **(E)**, the number of the platform location crossing** (F)**, the percent time the mice swam in the target quadrant **(G)**, the distance the mice swam in the target quadrant **(H)** and the swim speed **(I)** of the mice during the 60 s probe tests are shown. Data are presented as mean ± SEM (*n* = 12–15 mice per group).

We recently found that intranasal administration of insulin (1.75 U/day) for seven consecutive days can prevent anesthesia-induced impairment of spatial learning and memory in aged wild type mice (Zhang et al., 2016). We wondered whether intranasal insulin is also effective in preventing anesthesia-induced spatial learning and memory deficit in 3xTg-AD mice. In addition, we speculated that seven daily doses might not be needed for this prevention. Thus, we treated 7–8-month-old 3xTg-AD mice with intranasal insulin (1.75 U/day) daily for only three consecutive days before anesthesia and assessed the spatial learning and memory by MWM starting on the following day after the anesthesia. We found that the insulin treatment prevented anesthesia-induced spatial learning deficit, as evidenced by significantly less time needed for the insulin-treated group to locate the hidden platform during the training sessions (Figure 2A). The learning curve of the insulin-treated mice post anesthesia was actually overlapped with that of the control mice without anesthesia exposure. During the probe trial, the insulin-treated group also crossed the former platform location significantly more times than the untreated groups (Figure 2B), although the percentage of time and distance in the target quadrant were similar among the three groups (Figures 2C,D). We also performed reversal MWM test on these 3xTg-AD mice beginning on 1 day after the standard MWM probe trial. The insulin-treated group was found to find the new hidden platform significantly faster than the anesthesia group without prior treatment with insulin during the reversal training phase (Figure 2E). Prior treatment with insulin also significantly increased the percentage of time and distance traveled by mice in the target quadrant during the reversal probe trial (Figures 2F–H). The swim speeds of the mice were not different among the three groups (Figure 2I), which otherwise might interfere the water maze test. These results indicate that only three daily doses of intranasal insulin are sufficient to prevent anesthesia-induced cognitive impairment in 3xTg-AD mice.

### Anesthesia Induces Long-Term Neurobehavioral Changes in 3xTg-AD Mice

**Figure 3:**
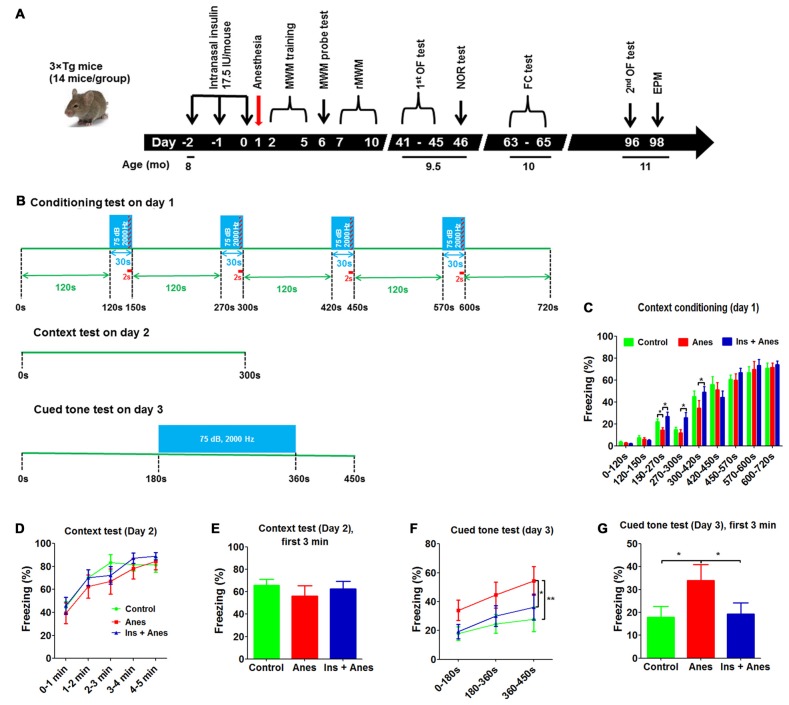
**Effect of anesthesia and intranasal insulin on long-term behavior of 3xTg-AD mice. (A)** Experimental design. The 3xTg-AD mice (7–8 months old, female) received daily intranasal insulin or saline for 3 days, followed, on the next day, by anesthesia with propofol/sevoflurane for 3 h. The mice were then tested using various behavioral tests at various time periods indicated in the diagram. MWM, Morris water maze; rMWM, reversal MWM; OF, open field; NOR, novel object recognition; FC, fear conditioning; EPM, elevated plus maze. **(B)** FC test schedule, which was carried out over three consecutive days. **(C)** Percentage of time the mice froze during the conditioning phase on day 1 of the FC test. **(D)** Percentage of time the mice froze in different time periods (data with 7 s bout) during the 5-min context test on day 2 of the FC test. **(E)** Percentage of time the mice froze during the first 3 min (data 7 s bout) in the context test. **(F)** Percentage of time the mice froze in different time periods (data with 7 s bout) during the cued tone test on day 3 of the FC test. **(G)** Percentage of time the mice froze (data with 7 s bout) during the first 3 min in the cued tone test results. Data are presented as mean ± SEM (*n* = 11–13 mice per group).

The long-term effect of general anesthesia on cognitive function remains elusive. Thus, after determining the acute effect of anesthesia on reference memory by MWM, we assessed the cognitive function of the 3xTg-AD mice from the above study on day 46 post anesthesia by using novel object recognition test, on day 63–65 by using fear conditioning test, and on day 98 by using elevated plus maze test (Figure 3A). Novel object recognition test, which assesses the short-term memory, did not show any significant differences between the mice exposed to anesthesia 46 days ago and the control mice (Supplementary Figure S2). However, fear conditioning test, which assesses the amygdala- and hippocampus-dependent memory, performed 63–65 days after exposure to anesthesia indicated changes in the anesthesia-treated mice (see below).

The mice were conditioned in a conditioning chamber for 12 min, and a 30-s tone was followed by a 2-s electric shock every 120 s (Figure 3B). We observed that the freezing time of the anesthesia-treated mice was significantly shorter than the control-treated mice during the 150- to 300-s conditioning (Figure 3C), suggesting an abnormality of associating the context/tone to the shock or an impairment in remembering the shock by these mice. The anesthesia-treated mice caught up with the control-treated mice after continued conditioning, suggesting that this long-term abnormality induced by anesthesia was mild and overcome with further training. Contextual fear conditioning test performed on the next day did not yield any significant difference between the anesthesia and the control groups (Figures 3D,E and Supplementary Figure S3). To our surprise, much longer freezing time was observed in the anesthesia-treated mice than the control-treated mice in the cued fear conditioning test performed on the third day (Figures 3F,G), suggesting that the anesthesia-treated mice might have developed some abnormality that made them “freeze” more during the cued fear conditioning test. Similar results were seen when various bouts were used for the data analyses (Supplementary Figure S4).

To study if the increased freezing time of the anesthesia-treated mice in the cued fear conditioning test could be due to reduced spontaneous activity or altered anxiety level, we analyzed the data of open field test performed before the novel object recognition test (Figure 3A). However, analyses of the total and central distance coverage, central area time and central area entries in the open field indicated no significant differences between the anesthesia-treated and control-treated groups (Supplementary Figures S5A,B). To eliminate a possibility that these neurobehavioral changes might occur after the open field test, we tested these mice again on day 96 post anesthesia and again found no significant differences in these parameters between the two groups (Supplementary Figure S5C). We also tested these mice in an elevated plus maze for anxiety after the second open field test and again found no differences between the two groups (Supplementary Figure S6). These results exclude the possibility that the increased freezing time of the anesthesia-treated mice in the cued fear conditioning test above was caused by reduced spontaneous activity or altered anxiety level.

### Intranasal Administration of Insulin Prevents Anesthesia-Induced Long-Term Neurobehavioral Changes in 3xTg-AD Mice

To investigate whether intranasal administration of insulin can prevent any of the detectable long-term neurobehavioral changes, the insulin treated group was also included for the long-term study (see Figure 3A). We found that three daily doses of intranasal insulin administrated before exposure to anesthesia prevented the mice from anesthesia-induced long-term memory impairment, as indicated by the complete prevention of the reduction of the freezing time of the anesthesia-treated mice during the 150- to 300-s conditioning (Figure 3C). The marked increase in the freezing time in the anesthesia-treated mice observed in the cued fear conditioning test was also prevented completely with insulin treatment (Figures 3F,G and Supplementary Figure S4). These results suggest that intranasal insulin treatment of mice for only 3 days before anesthesia can have significant effect on preventing anesthesia-induced long-term neurobehavioral changes. The intranasal insulin treatment also increased the number of open arm entries of the 3xTg-AD mice tested longer than 3 months after insulin administration (Supplementary Figure S6A).

## Discussion

Anesthesia increases the risk for cognitive impairment and may be the major cause of POCD (Hudson and Hemmings, 2011). Preclinical studies have demonstrated the role of various anesthetics in the development of neuropathological changes in the brain. However, little is known about what might impact the anesthesia’s role in POCD and how to prevent this adverse effect of anesthesia. In the present study, we found that both aging and the prior existence of AD-like brain pathology promote the cognitive impairment caused by anesthesia in mice. We found that under our well controlled experimental conditions, anesthesia impaired the spatial learning and memory of the aged, but not younger adult wild-type (WT) mice. Increased impairment of spatial learning and memory was observed in adult 3xTg-AD mice carrying early-stage AD-like brain pathology. Our findings are consistent with epidemiological studies showing that aged and vulnerable individuals are at increased risk for developing POCD (Chen C.-W. et al., 2014; Chen P.-L. et al., 2014; Patel et al., 2016; Sprung et al., 2016). Propofol and sevoflurane are among the commonly used combinations for anesthesia in the clinic. The use of these two anesthetics in the present study makes our study more clinically relevant.

Our findings show a clear role of aging in increasing the vulnerability of mice to anesthesia-induced cognitive impairment. Previous studies also suggested that animals of different ages may react to anesthesia very differently. Anesthesia with 1.2% isoflurane and 70% nitrous oxide for 2 h was shown to improve memory in younger rats (6 months old) but compromise it in old rats (18 months old; Culley et al., 2003). Repeated anesthesia with isoflurane (35 min daily for 4 days) induced persistent, progressive memory impairment, caused a loss of neural stem cells, and reduced neurogenesis in neonatal (14 days old) rats and mice, but not adult (60 days old) rats (Zhu et al., 2010).

There are only a few animal studies reporting the long-term cognitive impact of anesthesia, and the results of these studies are inconsistent. One study showed that halothane or isoflurane did not accelerate the cognitive impairment of the 6-month-old 3xTg-AD mice 2 months post anesthesia, although the hyperphosphorylation of tau in the hippocampus was obvious (Tang et al., 2011). Exposure of aged (18 months old), but not younger (6 months old), adult rats to a clinically relevant mixture of isoflurane and nitrous oxide for 2 h led to long-lasting impairment on a spatial-memory task that was learned by the animals before anesthesia administration (Culley et al., 2003). By contrast, neither younger nor aged animals showed impaired learning of a new spatial-memory paradigm following the above anesthesia protocol or sevoflurane exposure (Culley et al., 2004; Callaway et al., 2012). Anesthesia of aged animals with propofol is reported not to induce persistent memory deficits (Lee et al., 2008). In the present study using 7–8-month-old 3xTg-AD mice, we observed significant neurobehavioral abnormality determined by fear conditioning test performed 63–65 days after exposure to anesthesia. However, novel object recognition test, which assesses the short-term memory, did not show any significant differences between the mice exposed to anesthesia 46 days ago and the control mice. Our findings suggest that anesthesia can induce some aspects of mild neurobehavioral abnormality that can last at least 2 months post exposure to anesthesia in mice. Because this neurobehavioral abnormality is mild and limited to only some aspects of the cognitive function and can be detectable only under certain conditions, it is not surprising that some previous studies failed to find the anesthesia-induced long-term cognitive impairment in animal studies.

It is surprising that the anesthesia-treated 3xTg-AD mice froze for a longer time than the control-treated mice in the cued fear conditioning test, especially during the first 180 s of the test when no tone was induced. The underlying cause is not understood at present. However, we eliminated the possibility that the increased freezing time of the anesthesia-treated mice observed above in the cued fear conditioning test was caused by reduced spontaneous activity or altered anxiety level because neither was changed in these mice as determined both before and after the cued fear conditioning test. Nevertheless, our studies indicate that these neurobehavioral changes caused by anesthesia exposed more than 2 months ago can be prevented by prior intranasal administration of insulin.

Insulin is known to regulate neural development and neuronal activities and plays an important role in learning and memory (Ghasemi et al., 2013; Chen et al., 2014a, 2016). Drugs targeting insulin signaling appears to be promising in treating AD (Chen et al., 2016). Intranasal administration of insulin, which bypasses the blood-brain barrier and reaches the brain through several pathways including olfactory- and trigeminal-associated extracellular pathways and perivascular pathway, can avoid the side effects of peripheral administration of insulin (Dhuria et al., 2010). Intranasal insulin has been shown to enhance memory in both healthy adults and in AD patients (Benedict et al., 2004, 2007; Reger et al., 2008; Craft et al., 2012; Claxton et al., 2015). To investigate the possible molecular mechanisms by which intranasal insulin may improve memory and benefit AD patients, we treated 3xTg-AD mice with intranasal insulin in a recent study and found that the treatment restores insulin signaling, increases synaptic proteins, and reduces Aβ levels and microglia activation in the mouse brain (Chen et al., 2014c). Intranasal insulin also alleviates cognitive deficits and amyloid pathology by shifting the cleavage of APP toward the non-amyloidogenic pathway in a transgenic mouse model of AD (Mao et al., 2016). Hence, intranasal insulin probably helps the prevention of anesthesia-induced cognitive impairment through several molecular pathways. The insulin’s neuroprotective role against the anesthesia-induced abnormalities may be mediated through both insulin receptor-dependent and independent mechanisms. Future studies using insulin receptor knockout mice can help reveal the exact role of insulin receptor or insulin signaling in the anesthesia-induced cognitive impairment and whether or how much the neuroprotective role of intranasal insulin is through the insulin receptor.

Studies have shown that anesthesia can exacerbate Aβ pathology and induce hyperphosphorylation of tau in the brain (Perucho et al., 2010; Run et al., 2010), which may partially underlie the mechanisms by which anesthesia induces memory impairment and increases the risk for dementia and AD. Given that intranasal insulin can attenuate AD-like pathologies and improve the cognition of AD mouse models, we recently administered intranasal insulin to anesthesia-treated mice. We found that daily intranasal administration of insulin for 7 days attenuated anesthesia-induced hyperphosphorylation of tau in both 3xTg-AD mice (Chen et al., 2014b) and aged WT mice (Zhang et al., 2016). Furthermore, intranasal insulin also prevented the short-term cognitive impairment in aged mice (Zhang et al., 2016). These findings suggest that intranasal administration of insulin could be an effective way to prevent anesthesia-induced cognitive impairment. In the present study, we found that daily intranasal insulin for only three consecutive days before the mice were exposed to anesthesia is also effective in preventing the anesthesia-induced acute spatial learning and memory impairment in 3xTg-AD mice, as assessed by MWM on the days after the anesthesia. Furthermore, intranasal insulin seems to have long-term beneficial effect against anesthesia. We found that the anesthesia-induced changes in neurobehavior detected using fear conditioning test performed 2 months after exposure to anesthesia were prevented fully with prior three daily doses of intranasal administration of insulin. Thus, our findings suggest that three daily doses of intranasal insulin can prevent both the short-term cognitive impairment and long-term neurobehavioral changes induced by anesthesia.

It is worth noting that although insulin should had been cleared from the body within hours after administration, its protective effects were observed up to 2 months after insulin administration. It appears that insulin may modulate the brain in such a way that makes it more resistant to insults, like that induced by anesthesia, received right after insulin administration, so that the anesthesia’s long-term adverse effects can be prevented. It is actually not uncommon that some drug’s effects, either beneficial or detrimental, can last long after the drug is cleared from the body. For instance, anesthetics can induce detectable cognitive impairment 7–10 days after anesthesia, whereas the anesthetics are usually cleared from the body within hours.

In conclusion, by using WT mice with various ages and 3xTg-AD mice, we found in the present study that aging and prior existence of AD-like brain pathology make mice more vulnerable to anesthesia-induced cognitive impairment. Anesthesia of 3xTg-AD mice with propofol and sevoflurane for 3 h resulted in long-term (>2 months) neurobehavioral changes. Finally, we found that daily intranasal administration of insulin (1.75 U/mouse/day) for only 3 days prior to anesthesia can prevent the mice from anesthesia-induced both acute deficit in spatial learning and memory and long-term neurobehavioral changes. Our findings provide experimental data to support an effective yet simple treatment to prevent the risk of anesthesia-induced postoperative cognitive decline.

## Author Contributions

YC and C-XG designed research; YC, C-LD and ZW performed research; YC, C-LD and C-XG analyzed data; KI, FL and BZ participated in data analysis and intellectual discussion; YC and C-XG wrote the article; all authors commented and approved the final version of the manuscript.

## Conflict of Interest Statement

The authors declare that the research was conducted in the absence of any commercial or financial relationships that could be construed as a potential conflict of interest.
