# Supplementary material for: Intranasal Insulin Prevents Anesthesia-Induced Cognitive Impairment and Chronic Neurobehavioral Changes
Source: Front Aging Neurosci. 2017 May 10;9:136. doi: 10.3389/fnagi.2017.00136 (PMC5424543; doi:10.3389/fnagi.2017.00136)
Supplement: Supplementary file 1 [file Data_Sheet_1.pdf]

Intranasal Insulin Prevents Anesthesia-Induced Cognitive Impairment and Chronic Neurobehavioral Changes

Yanxing Chen[1,2]*, Chun-ling Dai[2]*, Zhe Wu[2,3], Khalid Iqbal[2], Fei Liu[2], Baorong Zhang[1#] & Cheng-Xin Gong[2#]

SUPPLEMENTARY MATERIALS AND METHODS

SUPPLEMENTARY TABLE 1. Primary antibodies used in this study.

Antibody	Type	Specificity	Phosphorylation sites	Source / Reference
R134d	Poly-	Tau		Tatebayashi et al., 2012 [1]
pT181	Poly-	P-tau	Thr181	Invitrogen, Grand Island, NY
pS199	Poly-	P-tau	Ser199	Invitrogen
pT212	Poly-	P-tau	Thr212	Invitrogen
pS214	Poly-	P-tau	Ser214	Invitrogen
pS396	Poly-	P-tau	Ser396	Invitrogen
pS400	Poly-	P-tau	Ser400	Invitrogen
pS404	Poly-	P-tau	Ser404	Invitrogen
pT422	Poly-	P-tau	Thr422	Invitrogen
12E8	Mono-	P-tau	Ser262/356	Dr. D. Schenk [2]
AT8	Mono-	P-tau	Ser202/T205	Thermo Fisher Scientific, Rockford, IL
PHF-1	Mono-	P-tau	Ser396/404	Dr. P. Davies [3]
Anti-GAPDH	Poly-	GAPDH		Santa Cruz Biotechnology

[1] Tatebayashi, Y., Iqbal, K. & Grundke-Iqbal, I. Dynamic regulation of expression and phosphorylation of tau by fibroblast growth factor-2 in neural progenitor cells from adult rat hippocampus. *J Neurosci* 19, 5245-5254 (1999).

[2] Seubert, P. *et al.* Detection of phosphorylated Ser262 in fetal tau, adult tau, and paired helical filament tau. *J Biol Chem* 270, 18917-18922 (1995).

[3] Greenberg, S. G., Davies, P., Schein, J. D. & Binder, L. I. Hydrofluoric acid-treated tau PHF proteins display the same biochemical properties as normal tau. *J Biol Chem* 267, 564-569 (1992).

SUPPLEMENTARY RESULTS

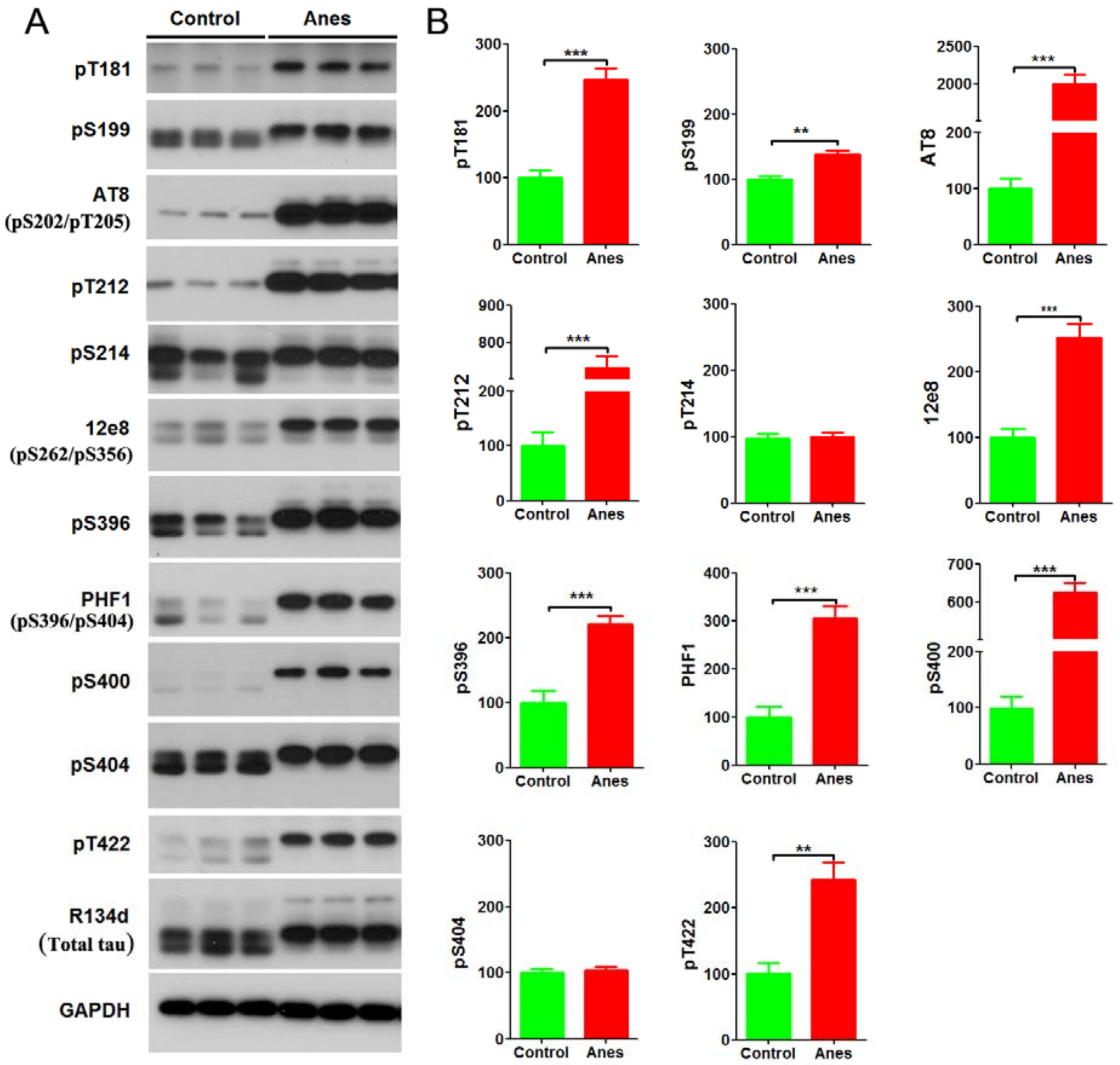

Supplementary Figure 1. Effect of anesthesia on tau phosphorylation in younger WT mice. (**A**) Homogenates of the forebrains of 7-8-month-old C57/B6-129 mice sacrificed at the end of anesthesia were analyzed by Western blots developed with antibody R134d against total tau and several phosphorylation-dependent and site-specific tau antibodies. Control group did not receive anesthesia. (**B**) Densitometrical quantifications (mean ± SEM) of the blots after normalization with the 134d blots. The levels of control group were set as 100. *$p < 0.05$ vs. control ($n = 6$ per group).

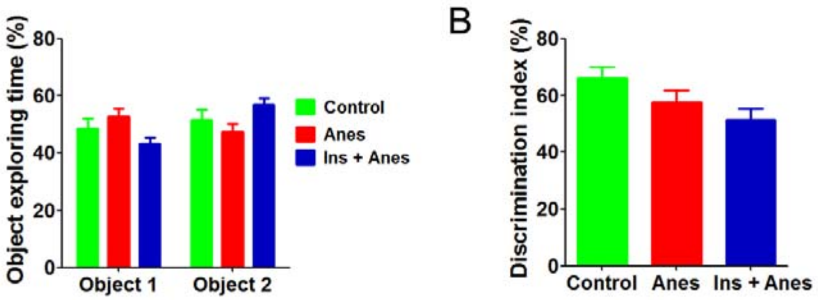

Supplementary Figure 2. Effect of anesthesia and intranasal insulin on short-term memory of 3xTg-AD mice tested using novel object recognition test. The 3xTg-AD mice (7-8 months old, female) received daily intranasal insulin or saline for 3 days, followed by anesthesia with propofol/sevoflurane for 3 hrs. The mice were then subjected to the novel object recognition test on day 46 post anesthesia exposure. (**A**) Percent time the mice spent exploring two identical objects during sample phase. (**B**) Discrimination index (the time exploring novel object divided by the total time exploring novel and familiar objects) in test phase. Data are presented as mean ± SEM (n=11-13 per group).

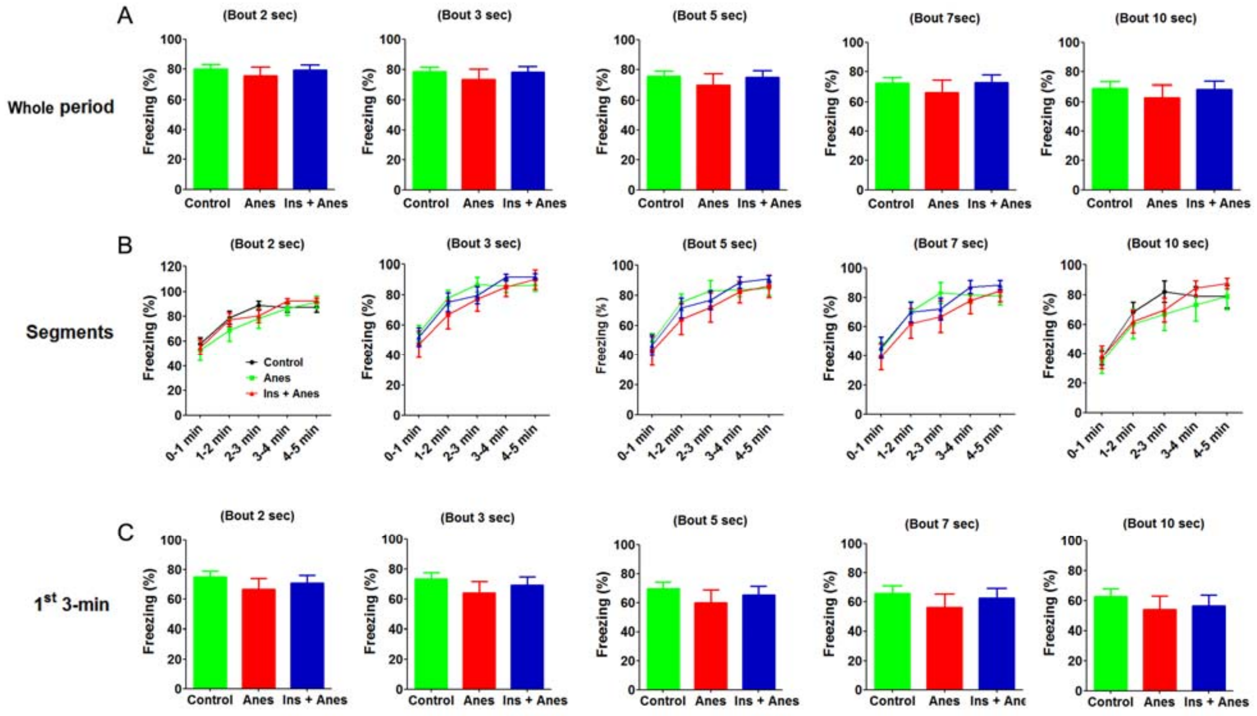

Supplementary Figure 3. Effect of anesthesia and intranasal insulin on long-term behavior of 3xTg-AD mice tested using the contextual fear conditioning test. The 3xTg-AD mice (7-8 months old, female) received daily intranasal insulin or saline for 3 days, followed by anesthesia with propofol/sevoflurane for 3 hrs. The mice were conditioned in the conditioning chamber for 12 min on day 63 post anesthesia exposure as shown in Fig. 3A, followed by testing on day 64 post anesthesia exposure. Data when various bouts were used for analyses are shown. (**A**) Percentage of time the mice froze during the 5-min context test. (**B**) Percentage of time the mice froze in different time periods during the 5-min context test. (**C**) Percentage of time the mice froze during the first 3 min context test. Data are presented as mean ± SEM (n=11-13 per group).

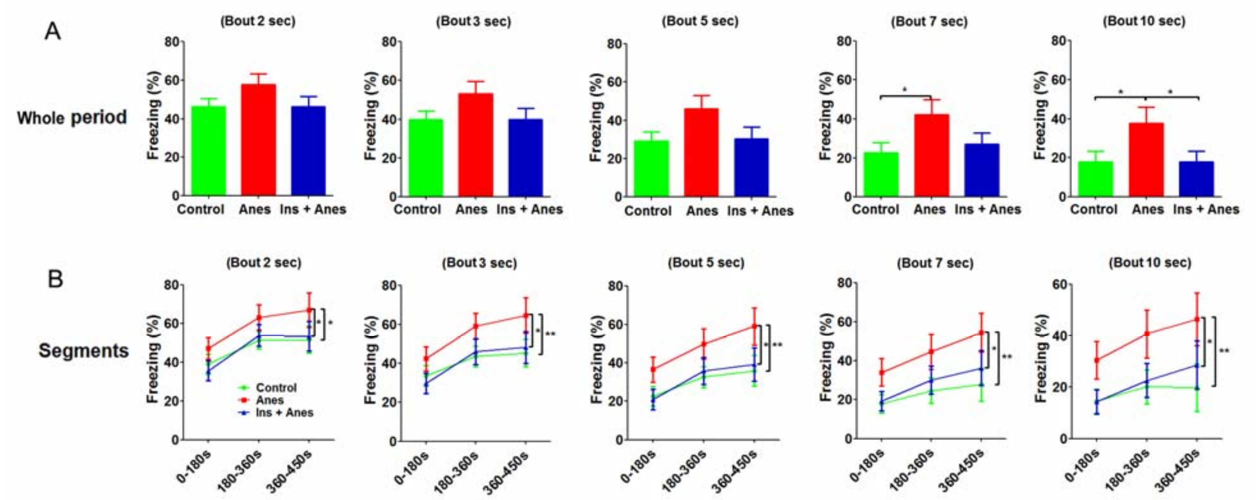

Supplementary Figure 4. Effect of anesthesia and intranasal insulin on long-term behavior of 3xTg-AD mice tested in cued fear conditioning test. The 3xTg-AD mice (7-8 months old, female) received daily intranasal insulin or saline for 3 days, followed by anesthesia with propofol/sevoflurane for 3 hrs. The mice were then tested using the fear conditioning test on day 63-65 post anesthesia exposure as shown in Fig. 3A and 3B. Data when various bouts were used for analyses are shown. (**A**) Percentage of time the mice froze during the 7.5-min cured tone test. (**B**) Percentage of time the mice froze in different phases during the 7.5-min cured tone test. Data are presented as mean ± SEM (n=11-13 per group).

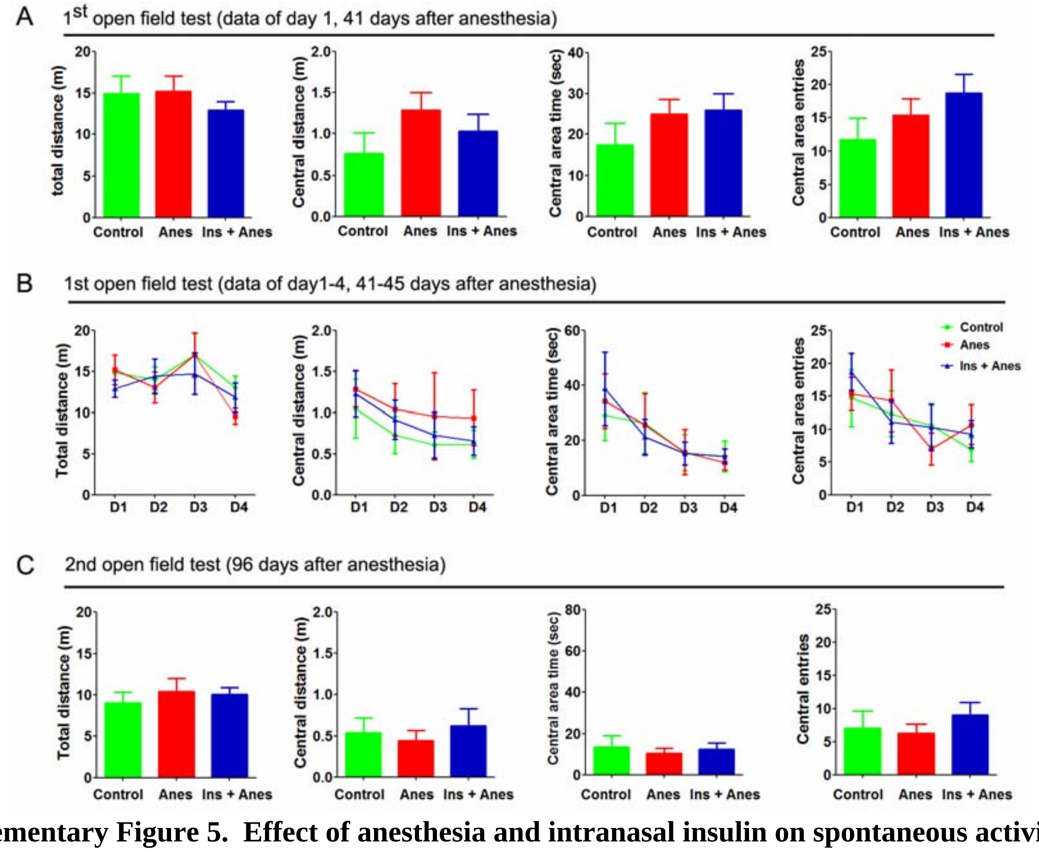

Supplementary Figure 5. Effect of anesthesia and intranasal insulin on spontaneous activity and anxiety of 3xTg-AD mice tested in an open field. The 3xTg-AD mice (7-8 months old, female) received daily intranasal insulin or saline for 3 days, followed by anesthesia with propofol/sevoflurane for 3 hrs. The mice were then tested in an open field for spontaneous activity and anxiety on day 41 (**A**), day 42-45 (**B**) and again day 96 (**C**) post anesthesia exposure as shown in Fig. 3A. Data are presented as mean ± SEM (n=11-13 per group).

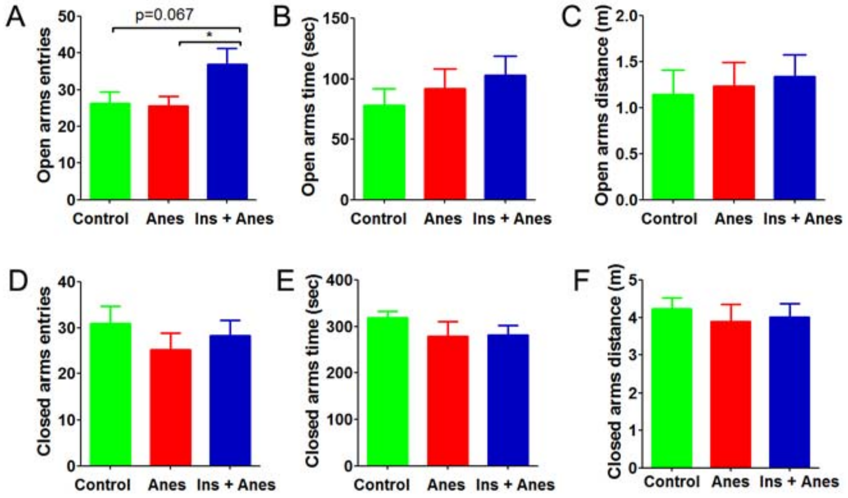

Supplementary Figure 6. Effect of anesthesia and intranasal insulin on anxiety of 3xTg-AD mice tested in an elevated plus maze. The 3xTg-AD mice (7-8 months old, female) received daily intranasal insulin or saline for 3 days, followed by anesthesia with propofol/sevoflurane for 3 hrs. The mice were then tested in an elevated plus maze for anxiety on day 98 post anesthesia exposure as shown in Fig. 3A. Data are presented as mean ± SEM (n=11-13 per group).
